# Group Dynamics in Automatic Imitation

**DOI:** 10.1371/journal.pone.0162880

**Published:** 2016-09-22

**Authors:** Ilka H. Gleibs, Neil Wilson, Geetha Reddy, Caroline Catmur

**Affiliations:** 1 Department of Psychological and Behavioural Science, London School of Economics and Political Science, London, United Kingdom; 2 College of Health and Wellbeing, University of Central Lancashire, Preston, United Kingdom; 3 Department of Psychology, King’s College London, London, United Kingdom; University of California Los Angeles, UNITED STATES

## Abstract

Imitation–matching the configural body movements of another individual–plays a crucial part in social interaction. We investigated whether automatic imitation is not only influenced by who we imitate (ingroup vs. outgroup member) but also by the nature of an expected interaction situation (competitive vs. cooperative). In line with assumptions from Social Identity Theory), we predicted that both social group membership and the expected situation impact on the level of automatic imitation. We adopted a 2 (group membership target: ingroup, outgroup) x 2 (situation: cooperative, competitive) design. The dependent variable was the degree to which participants imitated the target in a reaction time automatic imitation task. 99 female students from two British Universities participated. We found a significant two-way interaction on the imitation effect. When interacting in expectation of cooperation, imitation was stronger for an ingroup target compared to an outgroup target. However, this was not the case in the competitive condition where imitation did not differ between ingroup and outgroup target. This demonstrates that the goal structure of an expected interaction will determine the extent to which intergroup relations influence imitation, supporting a social identity approach.

## Background

In everyday life, people engage regularly in some sort of imitation, the involuntary mimicry of another person in terms of their movements, facial expressions, and emotions [1]. Imitation serves as a foundation for a beneficial social exchange and is described as one basic facet of social interaction [2–4]. That is, it is suggested that imitation makes smooth social interaction possible and serves as an important communicative tool that states that “I like you” [5, 6]. Here we investigate how intergroup and situational features of an interaction can modulate movement imitation.

### Behavioural coordination processes

The term ‘imitation’ refers to all situations where an observer performs the same action as that which they see, including both deliberate and involuntary copying of another person. It is the latter situation which we are concerned with in the present paper. Thus, we focus on the involuntary imitation of another’s actions in a social setting. Experimental social psychology mainly uses measures of imitation that are detected in naturalistic social situations (also termed ‘mimicry’). For example, Chartrand and Bargh [7] observed participants who had to describe pictures with a confederate. In one session the confederate repeatedly touched her face; in another, she moved her foot. Participants mimicked face-touching more than foot movement in the ‘face touching’ sessions and vice versa for the foot movement sessions (see [3] for a review).

Another aspect of behavioural coordination that is related to mimicry is synchrony, the tendency to perform repetitive actions (such as walking or rocking) at the same rate as another person. Although synchronized actions are not necessarily imitative (i.e. the observer does not always perform the *same* action as the other person), in most of the literature this term *does* refer to imitative actions, and thus we also review relevant studies on synchrony below. Synchrony, like imitation and mimicry, has been shown to be an important driver for (as well as consequence of) affiliation and interpersonal rapport [8–11].

Moreover, recently experimental psychologists have suggested that ‘automatic’ imitation [12–14] is a form of mimicry that can be studied in a controlled laboratory setting. Although limited work has been done to integrate the research on mimicry and automatic imitation, there is some evidence that the two concepts are equivalent [12; 15–16]. In particular, automatic imitation shares with mimicry the quality of being an involuntary tendency to copy another’s actions, and both mimicry and automatic imitation require the ability to match the visual representation of another’s action onto the observer’s own motor program. Automatic imitation can be measured using stimulus-response compatibility experiments in which both stimuli and responses comprise configural body movements. In such paradigms, the tendency to imitate others’ actions produces a stimulus-response compatibility effect in which the topographical features of task-irrelevant action stimuli facilitate similar, and interfere with dissimilar, responses [16]. In the present paper we investigate how automatic imitation is influenced by intergroup and situational features of an interaction. This is important because we know that features of the social environment (such as shared group membership) influence mimicry; what remains to be tested is whether we would observe similar effects on automatic imitation. That is, understanding whether such features modulate of automatic imitation in similar ways to mimicry would be an important step in bridging the mimicry and automatic imitation literature.

### Modulation of imitation

Although behavioural coordination is considered to be largely an automatic process, it is influenced by various facilitators and inhibitors including features of the social environment and the relationship between individuals who interact (see [1; 3], for an overview). For example, the desire to affiliate or create rapport leads to increased mimicry [3, 17]. Moreover, automatic imitation is greater when participants are primed with prosocial words than with antisocial words [15–16]. Generally, there is evidence for a social moderation of the tendency to imitate when we want to decrease social distance between individuals, create rapport and feel connected with others [18]. Much of the previous social psychological research has focused on the social moderators involved. That is, imitation depends on characteristics of the perceiver (mimicker) and the target (mimickee). We mimic friends more than strangers, and likeable ‘others’ more than unlikeable individuals [9, 19]. Several studies found that ingroup and outgroup distinctions influence imitation and that we are more likely to mimic ingroup members compared to outgroup members [20; 21]. However, in the domain of synchrony, Miles, Lumsden, Richardson and Macrae [9] showed that in an intergroup situation, participants who were supposed to interact with a member of an ostensibly different group (outgroup), displayed *more* synchrony than those who shared the same minimal group identity with the target. A similar effect whereby participants demonstrated increased imitation of outgroup members was recently found by Rauchbauer, Majdandzic, Hummer, Windischberger, and Lamm [22] Miles et al. argued that it might be the interplay between social category and the interaction setting that influences behavioural synchrony. They suggested that the presence of cooperation (i.e., an incentive to affiliate with a dissimilar other) during interpersonal coordination may act to buffer against intergroup differences and decrease social distance between members of different groups.

In the present work we extend this previous research in two ways. First, we establish how the social environment influences automatic imitation. The social psychological literature on naturalistic mimicry has found plenty of evidence for the social modulation of these effects. However, the question of whether social modulation also influences automatic imitation has been relatively neglected. Second, we focus on two elements of the social environment that influence imitation: we investigate how characteristics of the target affect imitation as a function of the goal structure of the social situation. More precisely, we investigate whether participants differ in their level of imitation of an ingroup or outgroup target depending on whether they expect they will need to cooperate or compete with the target person.

### Social relationship between perceiver and target in context

Our behaviour towards other group members largely depends on the environment in which an interaction occurs. According to the social identity approach (SIA, [23]) group memberships are not fixed and permanent but vary according to the context [24; 25]. The cognitive structure of the self is defined by multiple (social) categories (a woman, an academic, a daughter, fun-loving, German, charity supporter), which are hierarchically structured and become increasingly more inclusive. For example, when the category ‘academic’ is activated, one might distinguish between psychologists and sociologists and engage in intergroup differentiation and ingroup behaviour. When the category ‘friend’ is activated, however, behaviour is more likely to be guided by interpersonal relations between friends (which might include sociologists). Thus, the salience of a specific category helps to determine whom one perceives as an ingroup or outgroup member at a given point in time and when and how one engages in interpersonal or intergroup behaviour. In a relevant intergroup setting, interaction is determined by membership in social groups; we act in terms of our group memberships (as an academic, sports person, charity supporter) and motivations and behaviours towards a ‘target’ are based around our group memberships. Likewise, interpersonal relations and individual characteristics can determine individuals’ social behaviour when intergroup relations are less important and individual motivations are more pertinent [24]. Generally, we match social categories to properties of the social context and the category that is most ‘fitting’ will be salient in the given context; hence we cognitively match social categories to contexts.

As alluded to earlier, most previous research on imitation has focused on the main effects of group affiliation and very few studies have looked at how group membership is also influenced by the expected context (task relations, goal structures) in which individuals interact. An exception is the study by Miles and colleagues [9] that focused on the effect of the target’s group membership on synchrony. They provided preliminary evidence that not only group membership, but also participants’ expectations about a subsequent task, influence the level of behavioural coordination with an outgroup member. They revealed that stable coordination (i.e., in-phase synchrony) was more pronounced when participants interacted with a member of a different minimal group (outgroup-member) expecting a cooperative interaction situation, than when interacting with an ingroup member in the same situation. The authors argue that this increased synchronisation could be explained by a drive to decrease social distance with an ‘outgroup’ member prior to the expected cooperation; they hypothesized that individuals use synchrony to achieve an affiliation goal that is necessary for cooperation on the subsequent task, and that such interpersonal coordination might act as a buffer against intergroup differentiations. However, they did not explicitly test this explanation by varying the interaction situation systematically.

Taken together, we know that both intergroup membership and (dis-)affiliation goals can act as facilitators (inhibitors) for mimicry or imitation. It is less clear, however, how these facilitators act together. Do perceivers imitate more strongly when both ingroup membership and an affiliation goal are present? What happens when ingroup membership acts as a facilitator but at the same time a disaffiliation goal in terms of competitive goal structure is present (i.e. when competing with an ingroup member)? The aim of the present study is to extend previous work on social moderators of automatic imitation by examining the interplay of intergroup membership and the context in which such group membership occurs.

### Present study

In line with assumptions from the Social Identity Approach [24; 25], and earlier work on behavioural mimicry and imitation [20; 21], we expected that participants differentiate between ingroup and outgroup members and are more prone to imitate an ingroup member compared to an outgroup member. At the same time, we were also interested in the interplay of intergroup membership and the context (the goal structure) in which individuals interact. Early work on goal structures has differentiated between cooperative and competitive goals [26]. Goal structures are *cooperative* when goals of separate individuals are linked together and interdependent; on the contrary, goal structures are *competitive* when individuals are competing for an outcome and their goals are negatively linked [27]. Thus, a cooperative goal structure should trigger an affiliation goal whereas a disaffiliation goal is associated with a competitive goal structure.

In terms of the interplay between group membership and goal structure we would expect that under the competitive condition, imitation is low when the target is an outgroup member because both the target’s group membership and the anticipated goal structure of the task will produce a low motivation to affiliate or to decrease social distance. In addition, we expected that the competitive goal structure emphasizes interpersonal competition for participants interacting with an ingroup target and hence overriding the effect of shared group membership; hence if the participants interact with an ingroup member in a competitive situation, the situation is constructed as an interpersonal competition and therefore we also predicted low imitation for the ingroup target in the competitive situation.

For the comparison between an ingroup and outgroup target in the cooperation condition, the previous literature suggests two competing hypotheses. First, work on goal structures [26] as well as social identity [28] suggests that an affiliation goal should be especially strong when both group membership and the anticipated goal structure imply strong affiliation. That is, imitation should be facilitated both by the interaction with an ingroup target and the expected goal structure of the task; and in this case, imitation should be stronger for ingroup targets compared to outgroup targets under cooperation.

However, the previously reviewed research by Miles et al. [9] suggested that in a cooperative situation, imitation might serve as a means to reduce social distance and to increase interpersonal rapport with an outgroup target, predicting that imitation of an outgroup member will be stronger than imitation of an ingroup member.

In sum, we explored how the effects of social group membership and anticipated goals impact on the level of automatic imitation. We used a two (group: ingroup, outgroup) x two (situation: cooperative, competitive) design. The dependent variable was the degree to which participants imitated the other person (confederate) in a simple reaction time automatic imitation task (e.g. [29]), a relatively novel paradigm in the social psychological literature on imitation.

In addition to the measure of automatic imitation, we also included an explicit measure of affiliation: ingroup identification. We expected that participants would focus on an inter*personal* dimension when they anticipate competition with an ingroup member and hence we predicted decreased identification with the ingroup under the competitive, compared to the cooperative, condition. When participants anticipate competition with an outgroup member, we expected ingroup identification to be relatively high because the inter*group* context is salient. Thus, in line with our previous reasoning, we expected that both group membership and the anticipated goal structure would influence ingroup identification as a measure of group affiliation.

## Method

### Ethics statement

This study obtained ethical clearance from the University Ethics Committee at the University of Surrey (on 1 February 2012, EC/2012/07/FAHS) and from the Research Ethics Committee at the Department of Social Psychology at the LSE (on 4^th^ Oct 2013). Before completing the questionnaire, participants were informed about the aims of the study and participants gave written consent. After the study all participants were debriefed.

### Participants and design

Data were collected in two waves. Participants were 177 female students from two British universities (age: *M* = 20.83, *SD* = 3.60, range 18–48). Since the target was female, only female students were recruited, in order to avoid the need to include same/different gender of the perceiver/target as an additional intergroup variable. We only recruited right-handed participants because our target was right-handed. Eighty-one of the participants self-categorised as ‘white’ (45.7%) and 96 (54.3%) as ‘other’ (including Chinese, Asian, Black, Mixed); 48% were British and 52% not British and from 24 different countries. Since both the intergroup manipulation and the task to be completed during the anticipated cooperative/competitive situation depended on familiarity with British charities (see below), we measured familiarity with British charities as part of the debrief. Fifty-nine participants reported not being familiar with British charities and were thus excluded from the main analysis.

The present study consisted of a two (group affiliation: ingroup vs outgroup) x two (situation: cooperative vs competitive) between subjects design with automatic imitation as the dependent variable. Upon completion of the study participants received £5 or course credits as compensation.

### Procedure and materials

Participants arrived at the laboratory individually (wave 1) or in a group of four (wave 2). When participants arrived in a group of four, they were led to individual cubicles in the lab. These participants were also informed that they would each have to work with another participant in an adjacent cubicle at a later stage of the experiment. When participants arrived individually, another assumed participant (actually a female confederate) arrived shortly after, and was taken by the researcher to an adjacent lab. Participants were informed that they would subsequently be working with this other person at a later stage of the experiment. Hence participants in both waves of data collection were under the impression that they would be interacting with another person at some later point during the experiment, thus creating the precondition for anticipated cooperation or competition with another person.

Participants were then told that the investigation explored two separate and unrelated issues: (a) a study on attitudes and decision making in terms of support for charities, and (b) a reaction time task. First, a bogus charity preference test was administered in which a list of values were presented on a computer screen, and participants were required to report which values they preferred and were most important to them. After they had responded to 12 values, participants were informed that their responses had been analyzed by the computer program in order to calculate a ‘charity match’–a best fit between the individual’s preferences and the values embodied by a range of different charities. In reality, all participants received feedback from the computer that their value preferences best matched those of the British Heart Foundation charity. Participants were then given a wristband from the British Heart Foundation (BHF) to wear. They were told that this wristband pertained to a later part of the study.

After the bogus preference task, the experimenter told participants that they would subsequently have a conversation with the other participant. To manipulate one of our independent variables (nature of situation: cooperation vs competition), we informed participants that they would have to talk to that other participant in the adjacent lab/cubicle about charities (for a similar manipulation see [28]). Participants were randomly assigned to either the cooperation or competition situation. For the *cooperation condition* we informed participants that: “After the completion of the reaction time study, we invite you and another person to *discuss* how to support a charity most effectively. Here, we are interested in how *working with* another person affects decision-making. *The best joint idea* will be rewarded.” In the *competition condition*, they were told: “After the completion of the reaction time study, we invite you and another person to *debate* how to support a charity most effectively. We are interested in *how debating and contrasting* ideas affects decision-making. The *best idea* will be rewarded.”

In the next stage of the investigation, participants were asked to perform a reaction time task for an ostensibly separate study. It was explained that they would be doing a reaction time task via a webcam with the other person in the adjacent lab/cubicle, and whom they would later meet for the discussion about supporting charities.

**Fig 1:**
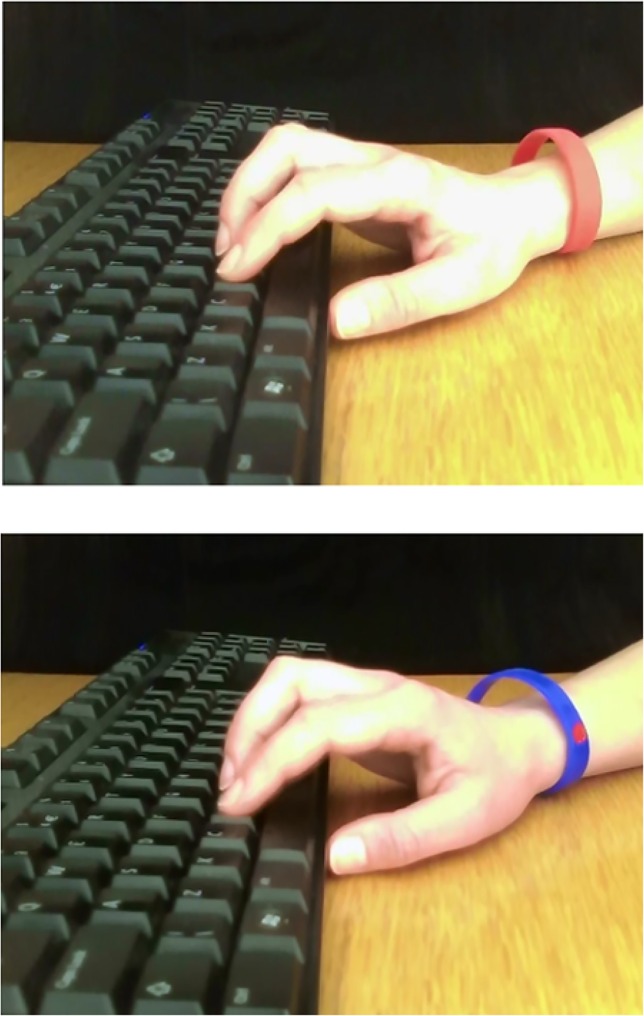
Ingroup (top) and outgroup (bottom) stimuli (single frames from the videos used in the ingroup and outgroup conditions).

Here, we manipulated our other independent variable (group affiliation target: ingroup vs outgroup), thus, whether participants would see over the webcam, and later have to cooperate/compete with, an ingroup or outgroup member. As each participant was given a wristband to identify their cause, they were able to see whether the other person on the webcam was an ingroup (wristband from BHF) or outgroup member (wristband from Poppy Appeal; see Fig 1). In reality, the webcam link was a video recording of the same female hand performing actions once with a BHF and once with a Poppy Appeal wristband. Participants were randomly allocated to either the ingroup or outgroup condition.

**Fig 2:**
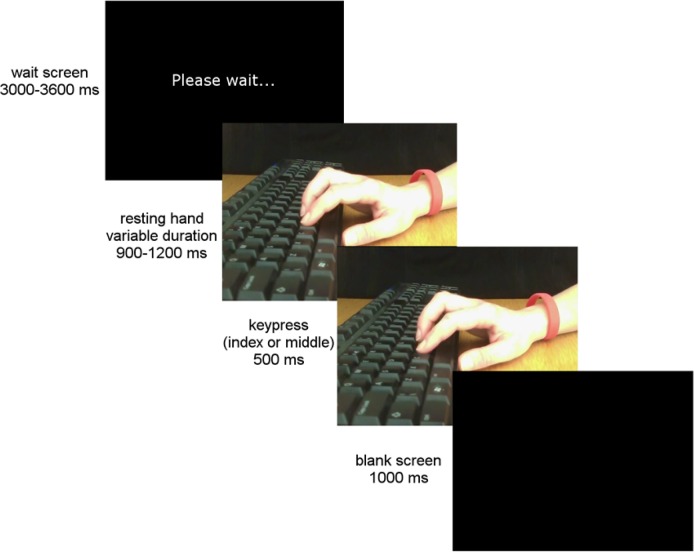
Example of trial structure.

The dependent variable was the degree to which participants imitated the other person (ostensibly the other participant; actually a video recording) in a simple reaction time imitation task (e.g. [29]). Participants’ response times to perform pre-specified key presses (using either the index or middle finger) were recorded. Participants were instructed to perform the pre-specified action (index or middle finger key-press) as soon as they observed the other person, supposedly via the webcam, perform a key press. Crucially, the video comprised recordings of both index and middle finger key-presses, performed in a random order. Thus response times to perform the same action as that observed, and to perform a different action to that observed, could both be measured. Imitation was calculated as the difference in response times when the participant performed a different action, compared to when they performed the same action, as that which they observed. In this paradigm an automatic imitation effect is generally found ([29, 11, 14] for a review) whereby participants are faster to perform the same action as that which they observe than to perform a different action, even when the observed action is task-irrelevant, as in the present case. The size of the imitation effect reflects the extent to which the observed action influences the observer’s action execution, and can be influenced by interpersonal factors relevant to the present study, such as the degree of pro-social orientation of the participant [15–16] (see Fig 2 and supplementary materials for further details on imitation task).

Following the imitation task, participants completed a short questionnaire. Specifically, as a check on the situation manipulation, they were asked to indicate on a 7-point scale what quality of face-to-face interaction they expected (1 = competitive; 7 = cooperative). Two items measuring *ingroup identification* were also included (“I feel similar to other people who support the BHF”; “I have a lot in common with other supporters of the BHF”; 1 = disagree, 7 = agree; *r*(99) = .74, *p* = .001). Lastly, participants were asked whether the other person was wearing the same or a different wristband and what they thought the study was about. All but one participant correctly reported the colour of the confederate’s wrist band. After participants answered some basic demographic information (age, ethnicity, and subject studied), they were fully debriefed about the nature of the study. In the debrief we questioned participants about features of the study; one question asked about familiarity with British charities and whether they had any pre-existing affiliation with British charities. This was to ensure that any lack of knowledge of British charities, or any pre-existing charity affiliations (i.e., if people were already affiliated with the BHF or Poppy Appeal charities) did not act as a potential confound.

## Results

### Checks and data screening

To determine our sample size, we conducted on a power analysis using GPower 3.1 [30]. We used the automatic imitation effect reported in Leighton et al. [16] as the basis for our power analysis. Given *d* = .86 and 80% power we would need a sample size of 18 per condition; however given the recent advice on sample sizes we aimed for a sample size around >25 per condition. This is a considerably larger sample size than reported in previous work on behavioural mimicry [7; 17] and in the automatic imitation literature [29; 12; 31]. 64 participants were included from wave 1 and 113 from wave 2. Nineteen participants were excluded from the main analysis because they made more than 20% errors on any of the three trial types, or had a mean response time more than 600ms (>2.58 standard deviations from the group mean), which left a sample of 158. However, wave 2 was conducted in a very international setting and in the funnelled debrief many participants said that they were not familiar with British charities and in particular the British Heart Foundation or the Poppy Appeal. As described above, 59 participants were not familiar with British charities and were excluded from the analysis. We therefore based our further analysis on the sub-sample of participants who were familiar with the charities. This resulted in a sample of 99 participants, 26 in the ingroup/cooperative condition, 24 in the outgroup/cooperative condition, 25 in the ingroup/competitive condition and 24 in the outgroup/competitive condition.

### Main analysis

#### Manipulation check

A univariate Analysis of Variance (ANOVA) was conducted with Group Affiliation (ingroup, outgroup) and Situation (cooperation, competition) as between-subjects factors on the item asking about the expected quality of interaction. One participant had a missing-value on the manipulation check leading to a sample of 98 for this analysis. There was a marginally significant main effect for situation, *F*(1,94) = 3.636, *p* = .06, η^2^ = .037^.^. Participants in the competition condition expected the interaction to be more competitive (*M* = 3.87, SD = 1.48) compared to participants in the cooperation condition (*M* = 4.45, SD = 1.50). Neither the main effect for group (*F*(1,94) = .60) nor the interaction (*F*(1,94) = .62) were significant, *ps*>.45.

#### Imitation

**Fig 3:**
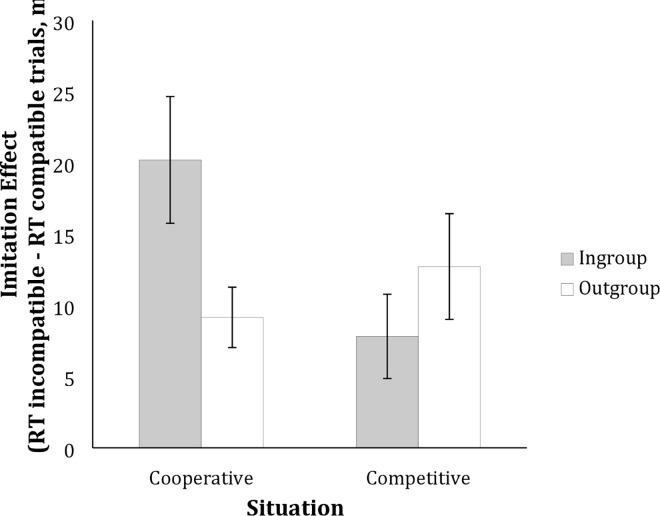
Mean ± standard error of the mean imitation effect in the four conditions.

Mean response times for correct responses on compatible and incompatible trials were calculated. The imitation effect was then calculated as the difference between response times on incompatible and compatible trials, and these values are displayed in Fig 3.

When conducting a 2 (Group Affiliation: ingroup, outgroup) by 2 (Situation: cooperative, competitive) ANOVA with imitation effect as the dependent variable we found that only the two-way interaction was significant, *F*(1,95) = 5.26, *p* = .024, η^2^ = .05. Neither the main effect for group affiliation, (*F*(1,95) = .78, *p* = .38, η^2^ = .008), nor for the situation (*F*(1,95) = 1.60, *p* = .21, η^2^ = .017) were statistically significant. We decomposed the interaction comparing cooperative vs competitive goal structure. When the situation was expected to be competitive, participants did not differ in imitation of an ingroup target (*M* = 7.81, *SD* = 14.49) compared to an outgroup target (*M* = 12.72, *SD* = 18.55), *M*Δ = 4.90, 95% [CI -14.72, 4.9], *p* = .32, Cohen’s *d* = .31. For cooperation, however, we see that the membership of the target matters: participants showed more imitation of an ingroup target (*M* = 20.21, *SD* = 22.69) compared to an outgroup target (*M* = 9.15, *SD* = 10.37), *M*Δ = 11.06, 95% CI [1.33, 20.78], *p* = .026, Cohen’s *d* = .63. Thus, when expecting a competitive goal structure, participants did not differ in terms of imitation regardless of their group membership. Imitation was strongest when cooperation was expected *and* the target was an ingroup member; thus, when intergroup membership and the expected situation were both associated with a strong affiliation goal.

#### Additional analysis

When we included familiarity with the charity as a covariate and conducted a 2 (Group Affiliation: ingroup, outgroup) by 2 (Situation: cooperative, competitive) by 2 (Charity Familiarity: familiar, unfamiliar) ANOVA with imitation effect as the dependent variable, only the three-way interaction was significant, *F*(1,149) = 7.106, *p* = .009, η^2^ = .046. When decomposing this effect, we found that the expected two-way interaction for Group Affiliation and Situation was not significant for those participants who were unfamiliar with the charities, *F*(1,54) = 2.46, *p* = .12, η^2^ = .044 but was for those who were familiar with the charities, *F*(1,95) = 5.26, *p* = .024, η^2^ = .052.

In addition, when we run this analysis separately for the two separate waves, we find the interaction (though weaker) for both samples: for wave 1 the effect was *F*(1,54) = 3.185, *p* = .080, η^2^ = .056 and for wave 2 it was *F*(1,37) = 2.860, *p* = .099, η^2^ = .072. This may reflect the lack of power to detect an effect in the smaller samples obtained in the two separate waves. Furthermore, if we consider automatic imitation as an imitative compatibility effect that suggests that the visual representation of an irrelevant movement stimulus is matched onto the observer’s motor representation of the same movement, we should see that observing an irrelevant movement stimulus facilitates responses on compatible trials and interferes with responses on incompatible trials. Hence, we can run a repeated-measures analysis of variance (ANOVA) with within-subjects factor of imitative compatibility (compatible, incompatible) and group affiliation (ingroup, outgroup) and situation (cooperative, competitive) as between-subjects factors. As expected the main effect for imitative compatibility was significant, *F*(1,95) = 51.41, *p* < .001, η^2^ = .35; response times were significantly faster for compatible than for incompatible distractor movements. In addition, the three-way interaction of imitative compatibility, group affiliation and situation, which mirrors the between-subject analysis on the imitation effect, was significant, *F*(1,95) = 5.26, *p* = .024, η^2^ = .052.

#### Ingroup identification

To test whether the manipulations had an effect on identification with the ingroup, we conducted a 2 Group Affiliation (ingroup, outgroup) by 2 Situation (cooperative, competitive) ANOVA with ingroup identification as the dependent variable. Only the interaction was significant, *F*(1,95) = 5.43, *p* = .022, η^2^ = .054. Neither the main effect for group affiliation, (*F*(1,95) = .55, *p* = .48, η^2^ < .005), nor for the situation (*F*(1,95) = .007, *p* = .98, η^2^ < .001) were statistically significant.

When decomposing the interaction comparing cooperation vs competition, we see that when the situation was expected to be competitive, participants’ ingroup identification was lower when the target was an ingroup member (*M* = 3.79, *SD* = 1.16) compared to an outgroup member (*M* = 4.44, *SD* = 1.11), *M*Δ = .648, 95% CI [.045, 1.25], *p* = .036, Cohen’s *d* = .57. That is, when a competitive goal structure is expected in an interaction with an ingroup member, ingroup membership is less relevant and ingroup identification is decreased, whereas when a competitive interaction is expected with an outgroup member, then ingroup identification is increased. However, for the cooperative situation, the membership of the target had no significant influence on the affiliation measure; participants’ ingroup identification was similarly high when the target was an ingroup member (*M* = 4.30, *SD =* 1.03) compared to an outgroup target (*M* = 3.95, *SD* = .93), *M*Δ = .35 95% CI [-.25, .947], *p* = .25, Cohen’s *d* = .35. Participants therefore seemed to distance themselves from the ingroup when competition was expected *within the group*, and/or increased their identification with the ingroup when competition was expected with an outgroup member. In a cooperative situation, in contrast, there is no requirement to alter ingroup identification as a function of the group membership of the anticipated interaction partner. Consistent with these results, for those participants in the ingroup condition, i.e. those who were imitating an ingroup rather than an outgroup member, there was a significant correlation between ingroup identification and the imitation effect, *r*(50) = .317, *p* = .025, such that imitation was largest for those participants who reported highest ingroup identification (note that it is not relevant to investigate this in those participants who were interacting with an outgroup member because we only measured their imitation of the outgroup and not of the ingroup).

## Discussion

The present results show that intergroup membership as well as the expected goal structure of an anticipated interaction act as social moderators for automatic imitation effects. Thus, we showed that the social environment in which social interaction happens influences automatic imitation: a result that mirrors similar research on naturalistic mimicry. Second, we found the first direct evidence that the facilitating effect of group membership varies according to expected goal structures: it is a combination of group membership and the expected goal structure that influences imitation. More specifically, we demonstrated that when the target was an ingroup member and participants expected cooperation, automatic imitation was the strongest. Under this condition two affiliation motives (ingroup membership and cooperation) were present that both fostered imitation. We also saw that identification with the ingroup as a more explicit measure of affiliation was high for anticipated cooperation with an ingroup member, as well as for anticipated competition with an outgroup member. Thus both cooperation with an ingroup member and competition with an outgroup member fostered ingroup identification.

However, for the ingroup-competition condition imitation was low. In this condition, group membership might have acted as a facilitator of imitation but the expected goal structure was an inhibitor, suggesting a shift from a (in-)group situation to an interpersonal situation, where participants focused on the competitive goal structure rather than their shared group membership [24]. This is also reflected in the finding that in the ingroup-competition condition, identification with the ingroup was lowest, and that ingroup identification was correlated with imitation of the ingroup.

Contrary to what Miles et al. [9] found, we did not see stronger imitation effects for an outgroup compared to an ingroup target under the cooperative condition. Imitation was significantly lower for an outgroup target than an ingroup target under cooperation. This suggests that in our study, the cooperative goal structure did not have the effect of decreasing social distance with an outgroup as theorised by Miles et al. Hence, we did not find any empirical support that introducing a cooperative goal structure reduced the perception of an intergroup setting and decreased social distance between perceiver and target. Our results are more in line with predictions from Social Identity Theory [24] and competing goal structures [28] than the empirical findings of this earlier study. However, in the present study we focused on imitation (see [20; 21]) rather than synchrony as in Miles et al. Although imitation and synchrony are related processes that have shared antecedences and consequences (for a review see [17]), the biggest difference is in the complexity of timing in synchrony, which is not apparent in imitation. Thus, the coordination that is inherent in synchrony requires much more anticipation of what the target is about to do than is necessary in imitation. This, together with the focus on the whole person as opposed to just a hand, might have contributed to the fact that participants in Miles et al.’s study focused more on commonalities than differences with the target, decreasing social distance even if they supposedly interacted with an ‘outgroup’ member.

### Limitations and future research

The present study had some limitations which we will address here. In order for the situation/goal structure variable to take effect, we attempted to create a precondition of an anticipated interaction (cooperative or competitive) with another supposed participant. It might have been that some participants were influenced by this more than others–some may have just focused on the immediate task at hand with little or no anticipation. However, our manipulation checks suggested that participants were at least cognisant of the nature of the anticipated interaction. Also, it could have been that some participants were not fully persuaded that they were interacting live via a webcam, although we did ask about such suspicions during the funnelled debrief and this was not an explicit issue. Finally, we chose to impose group memberships based on a bogus charity preference test performed by a computer program. As we have seen, this manipulation was not meaningful for participants who were not familiar with British charities and we excluded those from our analysis, which diminished power for our analysis and therefore dampens the strengths of our results. Thus, the group manipulation was not ‘minimal’, but depended on prior knowledge of the charities we used, as it appears that the social context we aimed to create only made sense to those participants who had some prior knowledge of the charities. This is an important constraint of our current work and needs to be addressed in future research. It is also important to understand in the light of recent debates in psychology and elsewhere highlighting the problems of insufficient power and small sample sizes [32, 33]. However, in the present case, the fact that the study did not have larger samples is a reflection of resource constraints and we encourage future research to replicate our results with a larger sample and a group manipulation that is relevant for a wider sample.

Another intriguing area for future research is to investigate the extent to which participants are aware of modulating their levels of imitation. We have shown that intergroup and situational factors interact to influence imitation, but what is not clear is the extent to which participants are using imitation as a conscious strategy to regulate social interaction or as an automatic response. It is possible that intergroup and situational factors influence imitation via low-level processes such as increasing attention to the target; via high-level conscious strategic processes, or via some other mechanism [15, 34; 35]. In line with this previous work we suggest that the modulating factors of group affiliation and goals influence affect the inhibition of the imitation effect. Yet, we need further research to explore this claim. Importantly, investigating the specific mechanism by which these factors act to influence imitation may also provide insight into improving social interaction in clinical disorders.

## Conclusion

The present results support the notion that imitation is influenced by multiple social context factors, such as intergroup membership but also the nature of an expected interaction. These results are in line with the notion that imitation is not a purely automated process that, once initiated, unfolds without being influenced by external factors, but rather that it depends on group dynamics in which individuals are embedded. With this, our results are in line with the idea that imitation has an important role in the regulation of social interactions with both ingroup and outgroup members. Our results provide a further bridge between the literatures on automatic imitation and mimicry, and suggest that imitation is a process that can signal group and interpersonal affiliation and subtly communicate affiliation or disaffiliation. Thus, humans are not just prone to (unknowingly) copy the actions of others: they do so with a message (of affiliation) that is congruent with the social context they are interacting in.

## Supporting Information

S1 Data (SAV)

S1 Data Syntax (SPS)

S1 File (DOCX)
