# Supplementary material for: Group Dynamics in Automatic Imitation
Source: PLoS One. 2016 Sep 22;11(9):e0162880. doi: 10.1371/journal.pone.0162880 (PMC5033460; doi:10.1371/journal.pone.0162880)
Supplement: S1 File — (DOCX) [file pone.0162880.s003.docx]

Supplementary materials: Detailed procedure for imitation task

Short digitized video sequences of a female hand performing index and middle finger key-presses on a keyboard (see Figure 2) were presented on a 17-inch screen using EPrime 2 (Psychology Software Tools). The video sequences ranged in length from 1400 – 1700 ms. Participants were seated about 80cm in front of the screen. The participant’s right hand rested on the keyboard.

In order to persuade participants that the reaction time experiment was an online webcam interaction with the other participant, and was taking place in real time, the following steps were taken. It was explained to participants that they were taking part in the reaction time experiment as a pilot for a brain scanning study, and that two participants were involved: a “sender” and a “receiver”. Participants were then informed that they would be the “receiver” and that their role was to perform pre-specified finger movements as fast as possible upon seeing the “sender” in the other lab perform a finger movement over the webcam. Prior to the first block, instructions appeared on the computer screen telling participants to wait until the webcam was set up and ready in the adjacent lab before continuing. A screen then appeared saying “waiting for sender” to give the impression of a real-time interaction, and after a ten second delay, a further screen saying “sender ready” appeared, indicating that the webcam was now switched on and the “sender” was ready to transmit to the participant’s computer.

The participant was then instructed which movement (index or middle finger keypress) they would be required to perform for the duration of the block. Participants were instructed to perform the instructed keypress irrespective of which movement was actually seen. They were also instructed that sometimes the other person would not make a movement at all, and that in that case they should not perform a keypress either. These “catch” trials, where no movement of the stimulus hand occurred, were included to ensure that participants were attending to the hand on the screen and not simply performing the instructed movement as quickly as possible.

The experiment was divided into four blocks of 40 trials per block. The instructed keypress was of the index finger (pressing the “v” key of the keyboard) for two blocks, and of the middle finger (pressing the “b” key) for the other two blocks. Block order was randomized in one of the following orders: index, middle, index, middle (imim; immi; miim; mimi). For each block, 16 compatible trials where the observed movement was the same as the instructed movement (e.g. index finger when index finger movements were instructed), 16 incompatible trials where the observed movement was different to the instructed movement (e.g. middle finger when index finger movements were instructed), and eight “catch” trials where no movement was observed, were presented in a randomized order.

Each trial commenced with the instruction “Please wait…” presented on the screen for a variable delay (3000 – 3600 ms). The video was then presented. Each video commenced with the hand resting on the keyboard. After a variable delay (900, 1000, 1100, or 1200 ms) the hand pressed either the “v” key with the index finger or the “b” key with the middle finger. The final frame of the keypress was frozen for 500 ms, after which the video ended. A blank screen was then presented for another 1000 ms so that participants’ response times could be recorded, after which the next trial commenced (see Figure 2). Response time was calculated as the elapsed time between the first frame of the keypress in the video, and the time at which the participant pressed either the “v” or “b” key. The videos were edited such that the first frame of the keypress occurred at exactly 900, 1000, 1100, or 1200 ms into the video, so that an accurate response time could be calculated.

The imitation effect is operationalized as a difference score between response times on incompatible (observed and executed movements are different) and compatible trials (observed and executed movements are the same), whereby higher values indicate stronger imitation (i.e. greater difference between incompatible and compatible trials) and lower values less imitation (i.e. smaller differences between incompatible and compatible trials).
